# Publishing for positive change in a turbulent world

**DOI:** 10.1007/s44217-022-00002-1

**Published:** 2022-05-10

**Authors:** Kerry J. Kennedy

**Affiliations:** The Education University of Hong Kong, Hong Kong, China

*Discover Education* is true to its name—it provides the opportunity for education researchers, policy makers and practitioners to share their insights, findings and ideas with global education communities. In this way, these communities can ‘discover’ how education can impact the multiple challenges that confront them.

The challenges are many. They range from a lack of equity in the provision of education for many of the world’s children to the promise of new pedagogies that can be inclusive of all students and the issue of new mental health conditions associated with disruptions to schooling as a result of COVID 19. The articles submitted to *Discover Education* will not on their own solve these problems, but they can contribute to the development of knowledge and skills that can contribute to solutions.

If you are thinking of submitting an article, *Discover Education* is particularly interested in education research and scholarship that has an impact outside of academic communities. I would like to see the work submitted here taken up by teachers or policy makers or parents and used to solve key problems. In this sense we need to move away from publishing for its own sake. The best publications should have a social purpose for the community and a mission to bring about positive change. This is a challenge for us, all but hopefully one that also will have benefits for many.

Welcome to *Discover Education*! We look forward to your submissions and the impact they will have in the life of your communities.

Professor Kerry J Kennedy.

The Education University of Hong Kong.

Editor-in-Chief.

*Discover Education.*

